# Power of the Plug Hat

**Published:** 1881-07

**Authors:** 


### POWER OF THE PLUG HAT.

The plug hat is virtually a sort of social guarantee for the preservation of peace and order. He who puts one on has given a hostage to the community for his good behavior. The wearer of a plug hat must move with a certain sedateness and propriety. He cannot run, or jump, or romp, or get into a fight except at the peril of his head-gear. All the influences of the beaver tend toward respectability. He who wears one is obliged to keep the rest of his body in decent trim, that there may be no incongruity between head and body. He is apt to become thoughtful through the necessity of watching the sky whenever he goes out. The chances are that he will buy an umbrella, which is another guarantee for good behavior, and the care of hat and umbrella—perpetual and exacting as it must ever be—adds to the sweetness of his character. The man who wears a plug hat naturally takes to the society of women, with all its elevated tendencies. He cannot go hunting or fishing without abandoning his beloved hat, but in the moderate enjoyment of croquet or lawn-tennis he may sport his beaver with impunity. In other words, the constant use of a plug hat makes a man composed in manner, quiet and gentlemanly in conduct, and a companion of ladies. The inevitable result is prosperity, marriage and church membership.

THOSE who have believed that the Sahara Desert is the bottom of a long dried-up sea, that it is considerably lower than the surface of the ocean, and that to convert it again into a big sheet of water all one has to do is to dig a canal, say to the Atlantic coast, had better read the lecture recently delivered in Paris by Dr. Lenz, who traveled through the desert from Morocco to Timbuctoo. He reports that the Sahara really forms a great plateau, which has an elevation of about 1,100 feet above the level of the Atlantic—a fact that makes the idea of flooding it ridiculous. There is nowhere a depression below the level of the ocean, and it is not a dead, sandy waste. There are rocks and sandy plains and oases covered with grass and stagnant sheets of water. Fresh-water fossils are found in abundance—another proof that it is not a dried-up sea. The climate, Dr. Lenz says, is not nearly as hot as he expected to find it; and wild beasts are rare, the most dangerous inhabitants being the tribes who, a short time since, massacred the French Trans-Sahara expedition. The doctor may be accused of "bulling" the Trans-Sahara railway scheme, which has been talked about a great deal of late in France, but as his story is also that of recent scientific explorers, the probabilities are that he does not exaggerate the good points of the desert.

